# Narrow locking compression plate vs long philos plate for minimally invasive plate osteosynthesis of spiral humerus shaft fractures

**DOI:** 10.1186/s12891-019-2757-z

**Published:** 2019-08-17

**Authors:** Jae-Jung Jeong, Sang-Eun Park, Hwan-Hee Lee, Jong-Hun Ji, Min-Sik Park, Yong-Taek Park

**Affiliations:** Department of Orthopaedic Surgery, Daejeon St. Mary’s Hospital, College of Medicine, The Catholic University of Korea, Seoul, 520-2, Deahung-Dong, Jung-Gu, Daejeon, 302-803 South Korea

**Keywords:** Minimally invasive plate osteosynthesis (MIPO), Spiral humeral shaft fractures, Narrow locking compression plate, Long philos plate

## Abstract

**Background:**

Our hypothesis was that minimally invasive plate osteosynthesis (MIPO) using long philos plate (LPP) would show better clinical and radiological outcomes and less complications than narrow locking compression plate (NLCP) for spiral humerus shaft fractures with or without metaphyseal fracture extension.

**Methods:**

From January 2009 to May 2016, we retrospectively studied 35 patients who underwent MIPO for spiral humerus shaft fractures with or without metaphyseal fracture extension (AO classification 12 A, B, C except A3). Eighteen patients underwent MIPO with a 4.5 mm NLCP (group I) in the early period of this study, while 17 patients underwent MIPO with LPP (group II) in the later period. Range of motion (ROM), pre- and post-operative anteroposterior (AP) and lateral angulation of the fracture, operation time, amount of bleeding, and functional outcomes including American Shoulder and Elbow Surgeons score, University of California at Los Angeles score, and Simple Shoulder Test score were analyzed at the final follow up.

**Results:**

All patients had complete bony union and achieved satisfactory functional outcomes except 2 patients. In LPP group, better outcomes in postoperative fracture angulation on X-ray and operation time (*p* < 0.05) were shown. But, two revision surgery with NLCP and bone graft was performed owing to 2 metal failures.

**Conclusions:**

In spiral humeral shaft fractures, LPP group showed better fracture reduction on X-ray and shorter operation time except metal failure owing to weak fixation. Even though MIPO technique using LPP is easier and more accurate reduction method, rigid fixation should be considered.

## Introduction

**Fig. 1:**
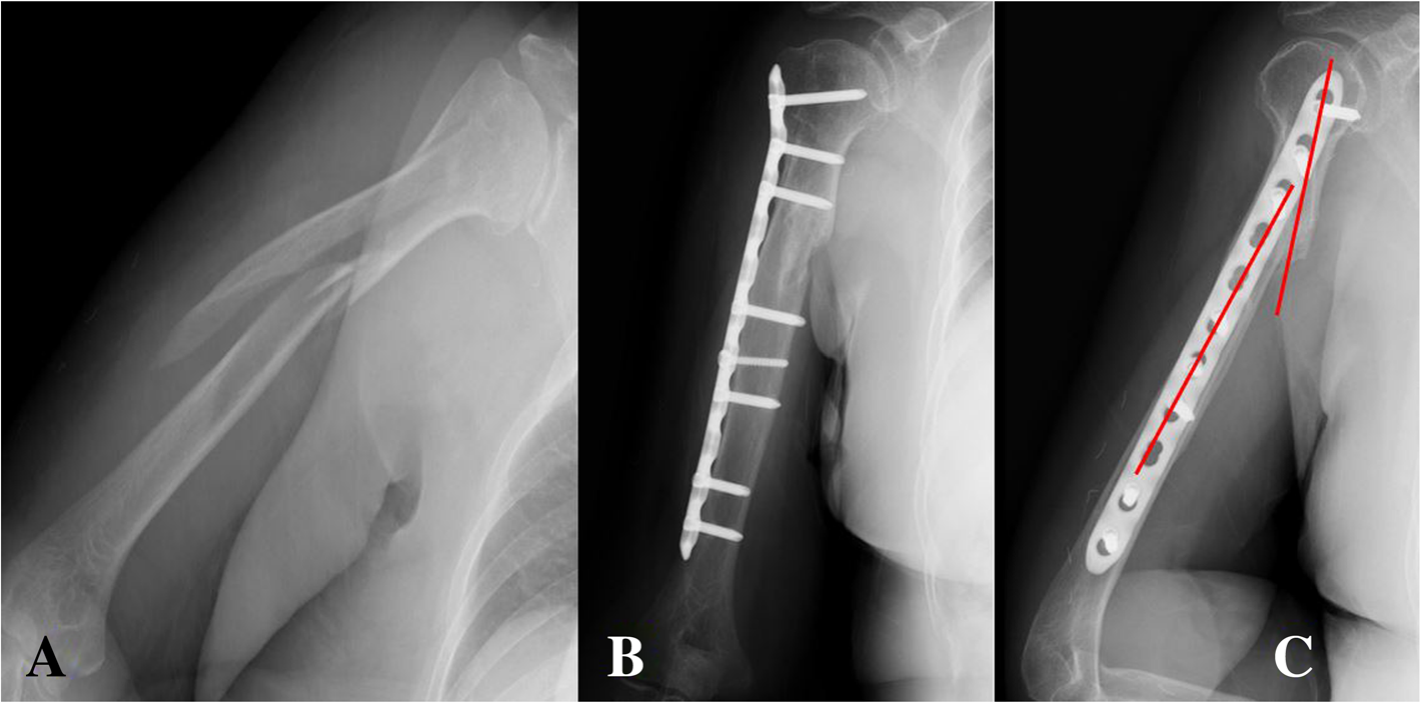
In MIPO technique using the narrow locking compression plate, intraoperative bending of this locking plate could adequately fitted to long spiral (Complex spiral type, AO classification: C1) humerus shaft fracture with proximal fracture extension to the greater tuberosity (**a**). However, at the postoperative lateral x-ray, the fracture angulation could be found after non-anatomical narrow locking compression plate fixation (**b**, **c**)

Minimally invasive plate osteosynthesis (MIPO) of humeral shaft fracture has been developed for the past 10 years and recently become popular treatment for these fractures. Conventional open plating has shown high bone union rates ranging from 88 to 100% [1]. However, due to its longer operation time and high infection rates (around 3–7%), less complicative operation methods was developed. MIPO technique for humeral shaft fractures has been developed to make stable fixation with less soft tissue dissection. It improves healing rates and reduces complications such as infection and iatrogenic radial nerve palsy. MIPO has consistently shown healing rates of more than 90%, even in open fractures [1]. However, there are some difficulties when using MIPO technique. In conventional open reduction and internal fixation (ORIF) technique, surgeons could see the fracture site easily and perform direct reduction using bone clamps or wirings. Indirect fracture reduction with traction and manipulation without full visualization of fracture site are only possible with MIPO technique [2]. One of the most challenging difficulties with MIPO technique is obtaining adequate reduction of fracture, especially humerus shaft fractures. Some surgeons often used the external fixator for temporary fracture reduction in the MIPO fixation. Anatomical bending is also a problem that needs to overcome, especially in humerus shaft fractures with proximal or distal farcture extension when using 4.5 mm narrow locking compression plate (NLCP, AO, Switzerland, Davos). In simple transverse fracture, it is enough to use a intramedullary nailing. However, in spiral humerus shaft fractures with proximal metaphyseal fracture extension, anatomically contoured bending of rigid NLCP plate to the proximal humerus is hard to make adequate fitted fixation (Fig. 1). It might require longer operation time with inadequate frature reduction. In MIPO technique using a long philos plate (long humeral locking plate), already manufactured anatomical plate can adequately fit for fractured humerus contour. It might provided easier reduction and less operation time than NLCP.

The purpose of this study was to compare clinical and radiological outcomes of patients using NLCP with those of patients using LPP for spiral humerus shaft fractures with or without metaphyseal extension. Our hypothesis was that a LPP for these fractures would show better clinical and radiological outcomes than NLCP.

## Materials and methods

Between March 2009 anf March 2017, 35 patients for whom MIPO technique was performed for spiral humerus shaft fractures with or without distal or proximal metaphyseal fracture extension were enrolled. All these spiral humerus shaft fractures were included in AO classification 12 A, 12 B and 12 C except 12 A3. These transverse humeral shaft fracture (12 A3) were excluded and these fractures coud be easily managed with intramedullary nailing. Sometimes, spiral humerus shaft fractures had metaphyseal extension and these fractures were extended to the greater tuberosity or distal humeral metaphysis area. At this time, narrow locking compression plate (NLCP) should be bent to match the contour of the humeral metaphysis.

Minimal invasive plate osteosynthesis (MIPO) using NLCP or long philos plate (LPP, AO, Switzerland, Davos) was performed consecutively for these spiral humerus shaft fractures. The introduction time of this LPP was later than NLCP in Korea and we could use this LPP in the later period of our study. We used conventional NLCP in the early period and then used LPP consecutively in these fractures. For the techniques had been performed by one senior shoulder surgeons with more than 15 years of experience in the field, there wasn’t significant learning curve with this technique. In the early period of this study, 18 patients underwent MIPO with 4.5 mm NLCP (Group I). And then, 17 patients underwent MIPO with LPP (Group II) consecutively in the later period. We reviewed patients’ charts including operation record, progression note, demographics, and preoperative or postoperative anteroposterior (AP) and lateral X-ray. Range of motion (ROM), preoperative and postoperative fracture angulation, operation time, amount of bleeding, and functional outcomes were analyzed at the final follow up and also were compared between the two groups. Range of motions were checked at 3 months, 6 months, and 1 year after operation. Clinical scores including American Shoulder and Elbow Surgeons (ASES) score, University of California at Los Angeles (UCLA) score, and Simple Shoulder Test (SST) score were check at 6 months and 1 year after operation. Patients’ demographics are shown in Table 1.

**Table 1:** Demographics of patients

		Group I (Narrow LCP^a^:NLCP)	Group II (long Philos plate: LPP)
Number		18	17
Age		62.7	61.2
Sex		M/F: 7/11	M/F: 4/13
BMI		24.4	25.9
Preop. AP angulation (mean value)		12.2°	12.4°
Preop. Lateral angulation (mean value)		6.96°	6.56°
Bone union		All union	15 union (2 metal failure)
complications		Radial nerve injury: 1	Radial nerve injury: 1
Injury mechanism	Fall down	3	0
	Traffic accident	4	7
	Work	0	1
	Blunt trauma	11	9
Fracture type	Closed	17	16
	Open	1 (type I)	1 (type II)
	AO classificaton	A: A1, A2, A3 = 5:3:0 B: B1, B2, B3 = 5:2:0 C: C1, C2, C3 = 3:0:0	A: A1, A2, A3 = 5:2:0 B: B1, B2, B3 = 3:1:1 C: C1, C2, C3 = 4:0:1

LCP^a^: locking compression plate

### Surgical technique

Under general anesthesia, beach chair position was used. Patient was placed on a conventional table in supine position. For MIPO technique using NLCP, a standard deltopectoral approach or transdeltoid approach (about 5 cm incision) was used for the proximal incision. The plate was inserted percutaneously from proximal to distal direction. Using a blunt elevator, submuscular tunnel was prepared. The length of the plate was adjusted to the fracture site and the location of the distal incision was determined under fluoroscopy. For the distal incision, anterior approach between biceps tendon or biceps and brachioradialis muscle was used [3, 4]. In this incision, radial nerve was not explored. Sometimes, these fractures were extended to the greater tuberosity or distal humeral metaphysis area and NLCP was bent to match the contour of the humeral metaphysis.

**Fig. 2:**
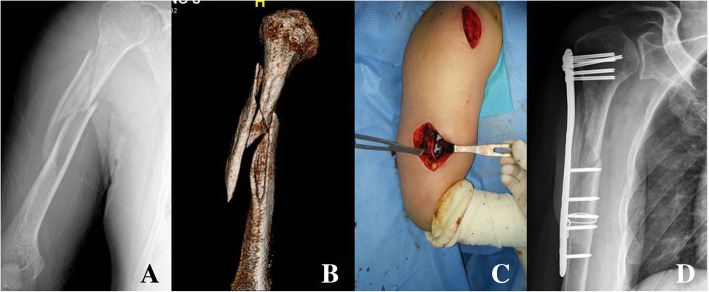
A 55 year-old male complained painful swelling of the upper arm after trauma. Conventional X-ray showed long spiral (Spiral wedge type, AO classification: B1) humerus shaft fracture with fracture extension to the proximal humerus (**a**, **b**). When using MIPO technique with long philos plate, the radial nerve was identified through the distal incision (**c**). Long humeral philos plate could fix this comminuted humeral shaft fracture adequately (**d**)

**Fig. 3:**
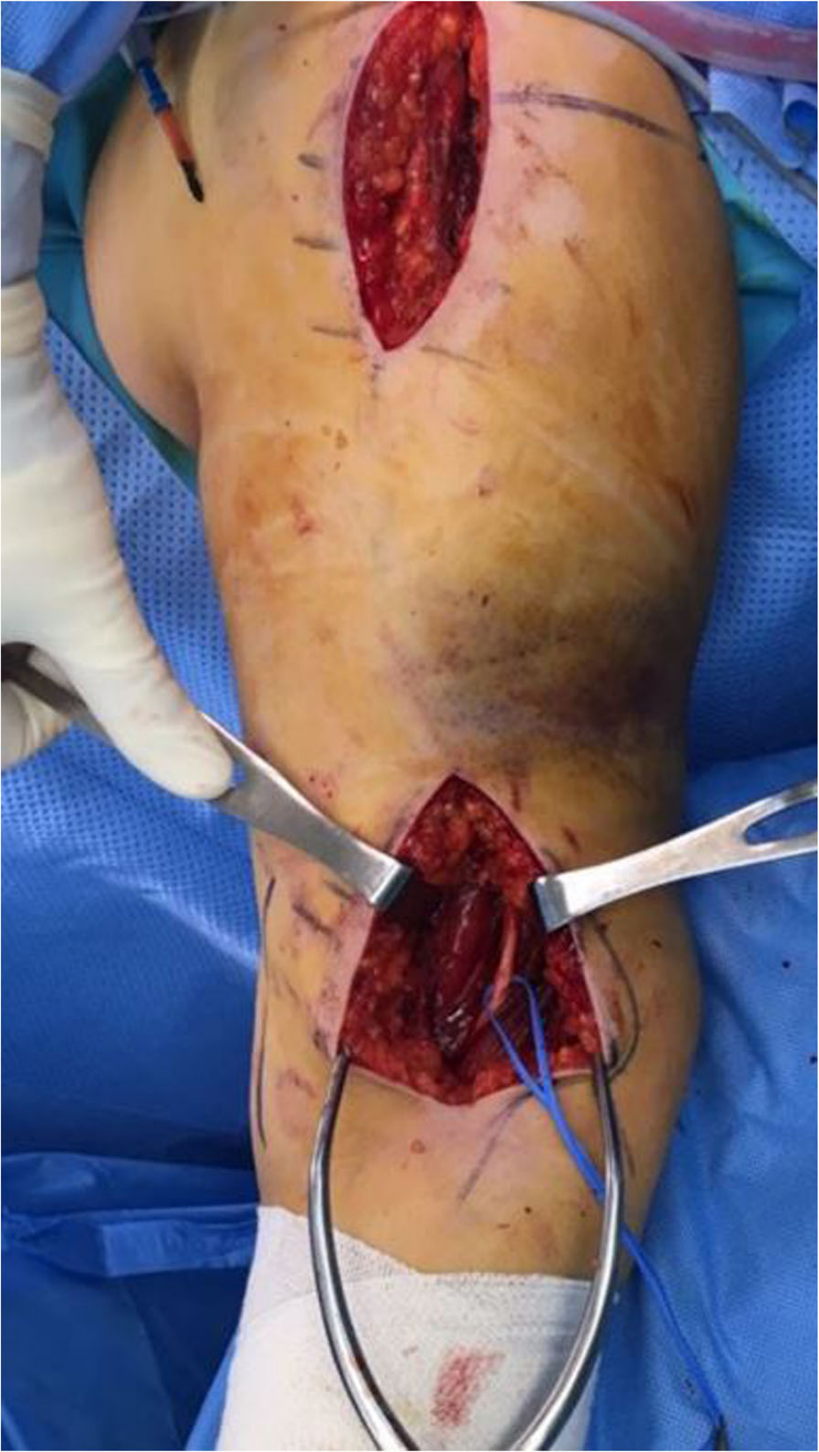
Through the distal incision, the radial nerve was identified in the anterior muscle compartment between brachialis and brachioradialis muscles. Repeated radial nerve exploration made us to reduce the time of radial nerve exposure dramatically

For the MIPO technique using LPP, linear 5 cm incision just below the acromion was performed for the proximal incision. The whole procedures were similar to the MIPO technique using NLCP except the proximal and distal incision. For the distal incision, the interval between brachialis and brachioradialis was dissected and then the radial nerve around the distal humerus was found (Fig. 2) [5]. To reduce the risk of radial nerve injury, the forearm was kept in full supination during this fascial incision. Through an oblique incision on the lateral aspect of the distal humerus as described by Livani et al. [6], the radial nerve was completely identified in the anterior muscle compartment between brachialis and brachioradialis muscles and followed proximally through the intermuscular septum which was released (Fig. 3). Excessive force was avoided during retraction of the lateral half of the brachialis muscle together with the radial nerve in the distal incision [5]. Repeated trial of radial nerve exploration made us to reduce the operation time of radial nerve exploration dramatically. If these spiral fracture was confined to the proximal part of the humerus shaft, radial nerve exploration didn’t explored and distal incision was confound to the middle humerus.

**Fig. 4:**
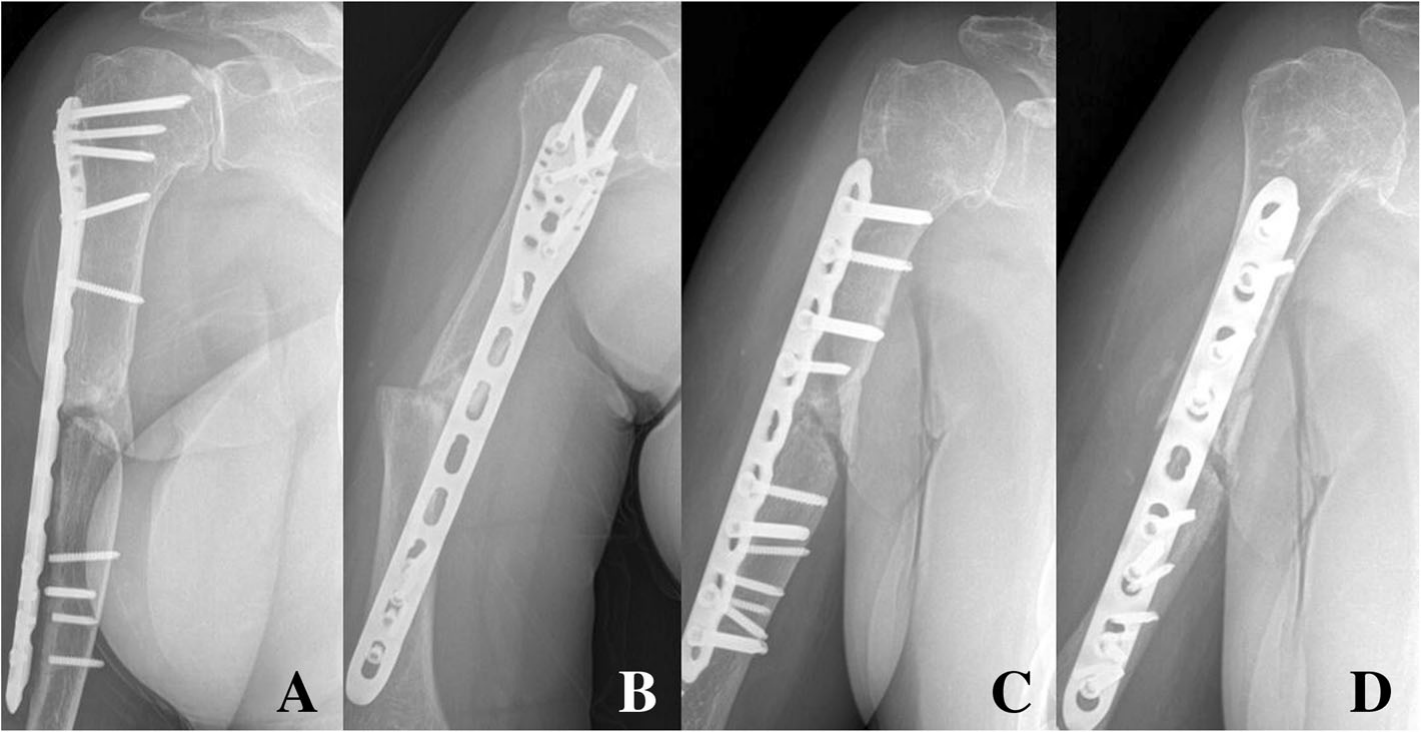
Anteroposterior and lateral X-ray showing the metal failure of spiral (Spiral wedge type, AO classification: B1) humerus shaft fracture of in a 74-year-old female patient (**a**, **b**). Using open approach, narrow locking compression plate was used with bone graft (**c**, **d**)

**Fig. 5:**
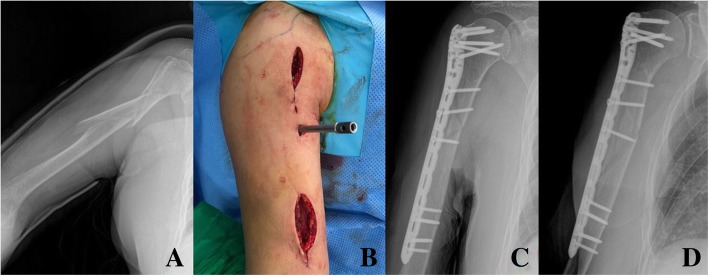
In a 85 year-old female patients after slip down injury, X-ray showed long spiral (Complex spiral type, AO classification: C1) humerus shaft fracture with proximal fracture extension to the greater tuberosity (**a**). In the midportion of the long philos plate, additional locking or compression screws were inserted through several small incisions for further rigid fixation (**b**). Long humeral philos plate could fix this complex humeral shaft fracture adequately (**c**, **d**)

Initially, K-wires were inserted to the uppermost hole of plate for temporary fixation of this LPP plate under C-arm guidance. Then we applied distal part of the long plate to the distal humerus while protecting the radial nerve. Compression screw in the uppermost proximal compression hole was inserted so that this anatomical LPP could reduce the fracture displacement easily. Consequently, compression screw in the distal part of the plate was inserted. If long oblique fracture was present or middle part of the fracture was unstable after proximal and distal compression screw insertion, additional locking or compression screw was inserted by using locking or compression drill sleeves at the middle part of the plate. In the earlier period of LPP group, no additional locking or compression screw at the middle part of the LPP plate developed two metal failure. After 2 failure, we always tried to insert additional positional screw in the middle part of plate of the unstable fractures (Figs. 4, 5). Step by step, proximal or distal locking screw were inserted. Finally, the status of fracture reduction was confirmed using fluoroscopy. Postoperatively, early ranges of motion of the shoulder and elbow joint was started immediately.

## Results

Mean follow-up was 16 months (range, 8 to 60 months). 33 patients had complete bone union and 2 patient who had failed with initial surgery using LPP achieved complete union with revision surgery using NLCP and bone graft. There were no other postoperative complications such as infection or non-union. Most patients had satisfactory restoration of shoulder function. Bony union was achived at a mean of 14 weeks (range, 10–18 weeks).

In 4.5 mm NLCP group, postoperative functional scores were improved from UCLA score of 26.0, ASES score of 77.1, and SST score of 7.4 at postoperative 6 months to UCLA score of 27.4, ASES score of 84.0, and SST score of 7.9 at the last follow up. The average operation time was 128.3 min and the amount of blood loss was 232.7 cc. AP and lateral angulations of fracture on x-ray view were improved after surgery (from about preoperatively 12°, 7° to postoperatively 5°, 6° at the final follow up).

In the LPP group, postoperative functional scores were improved from UCLA score of 25.1, ASES score of 57.8, and SST score of 5.1 at postoperative 6 months to UCLA score of 29.8, ASES score of 83.5, and SST score of 8.0 at the last follow up. The average operation time was 99.3 min and the amount of blood loss was 178.5 cc. AP and lateral angulations of fracture on x-ray were improved after the surgery (from preoperatively 12.4° and 6.56° to postoperatively 2.22° and 2.70° at the final follow up).

In our study, radiologic findings of both groups showed complete bony union except 2 patients. And those 2 patients achieved bony union after revision surgery. Their preoperative angulations in AP and lateral X-ray view were corrected significantly. In comparison with the NLCP group, LPP group showed higher correction of AP and lateral angulation significantly (*p* = 0.015 and *p* = 0.017, respectively). Furthermore, the LPP group had significantly better results in operation time than the narrow LCP group. (*p* = 0.025) But, There was no statistically significant difference in blood loss between 2 groups (Table 2). All clinical outcomes including ASES, UCLA, SST scores, and ROM were improved postoperatively in both groups. And, also, differences in clinical scores between the two groups were not statistically significant at postoperative 6 months or 1 year between the two groups. Mean postoperative ROM (forward flexion, abduction, external rotation and internal rotation) were improved in both group at every follow up. At postoperative 3 and 6 months, external rotation score in the NLCP group had better results than that in the LPP group (*p* = 0.020 and *p* = 0.023 at 3 and 6 months, respectively). (Tables 3, 4). However, only the degree of external rotation showed statistically significant difference [*p* = 0.020 (at 3 months) and *p* = 0.023 (at 6 months), respectively] However, there was no statistically significant difference in ROM at postoperative 1 year between the two groups (*p* > 0.05) (Table 5).

**Table 2:** Radiologic and operative findings at the last follow up for spiral humerus shaft fractures with proximal or distal fracture extension

	Group I (Narrow LCP^a^)	Group II (Long Philos plate)	*p*-value
Postoperative X-ray (Anteroposterior) (°)	5.14 ± 3.89(0.3–14.1)	2.22 ± 1.90(0–5.2)	0.015
Postoperative X-ray (lateral) (°)	5.91 ± 4.71(0.4–18.1)	2.70 ± 2.13(0–6.8)	0.017
Operation time (min)	128.3 ± 43.5(70–210)	99.3 ± 24.8(70–156)	0.025
Blood loss (cc)	232.7 ± 173.5(10–550)	178.5 ± 156.2(50–500)	0.368

LCP^a^: locking compression plate

**Table 3:** Range of motion at postoperative 3 months for humerus shaft fractures with proximal or distal fracture extension

	Group I (Narrow LCP^a^)	Group II (Long Philos plate)	*p*-value
Postoperative FF (°)	137.5 ± 21.2(100–170)	121.0 ± 16.1(100–150)	0.066
Postoperative Abd (°)	135.5 ± 21.5(100–170)	119.5 ± 14.6(100–145)	0.068
Postoperative ER (°)	40.0 ± 24.5(10–90)	17.0 ± 13.4(0–50)	0.020
Postoperative IR (level)	T12(L3-T7)	L2(L5-T8)	0.053

LCP^a^: locking compression plate, *FF* forward flexion, *Abd* abduction, *ER* external rotation, *IR* internal rotation

**Table 4:** Clinical outcomes at postoperative 6 months for humerus shaft fractures with proximal or distal fracture extension

	Group I (Narrow LCP^a^)	Group II (Long Philos plate)	*p*-value
Postoperative FF (°)	140.0 ± 21.5(100–170)	133.1 ± 8.8(120–150)	0.410
Postoperative Abd (°)	141.0 ± 20.2(100–170)	131.3 ± 10.6(115–150)	0.237
Postoperative ER (°)	44.4 ± 22.8(20–90)	21.9 ± 11.3(5–50)	0.023
Postoperative IR (level)	T12(L5-T7)	L1(L5-T7)	0.569
UCLA	26.0 ± 6.7(14–35)	25.1 ± 6.4(17–35)	0.787
ASES	77.1 ± 18.3(34–100)	57.8 ± 28.6(18–100)	0.113
SST	7.4 ± 2.2(2–10)	5.1 ± 3.7(0–10)	0.135

*LCP*^a^ locking compression plate, *FF* forward flexion, *Abd* abduction, *ER* external rotation, *IR* internal rotation. *UCLA* University of California at Los Angeles scale, *ASES* American Shoulder and Elbow Surgeons score, *SST* Simple Shoulder Test score

**Table 5:** Clinical outcomes at postoperative 1 year for humerus shaft fractures with proximal or distal fracture extension

	Group I (Narrow LCP)	Group II (Long Philos plate)	*p*-value
Postoperative FF (°)	154.1 ± 18.1(110–170)	143.0 ± 16.0(120–160)	0.261
Postoperative Abd (°)	155.0 ± 20.0(105–170)	140.0 ± 13.7(120–155)	0.153
Postoperative ER (°)	55.0 ± 20.1(20–90)	34.0 ± 11.4(20–60)	0.052
Postoperative IR (level)	T10(L2-T5)	T12(L3-T9)	0.094
UCLA	27.4 ± 6.9(15–35)	29.8 ± 3.3(25–33)	0.471
ASES	84.0 ± 9.6(60–96)	83.5 ± 18.0(56–100)	0.947
SST	7.9 ± 2.6(3–12)	8.0 ± 2.4(6–12)	0.948

Postoperative transient radial nerve injury was found in 3 patients (2 patients in NLCP group and 1 patient in LPP group) due to traction or retractors. These symptoms wtere completely recovered within 3 months. There was no infection in any cases.

## Discussion

The most important finding of the present study for spiral humerus shaft fractures with or without metaphyseal fracture extension was that LPP group showed better outcomes in postoperative fracture angulation on X-ray and operation time except metal failure. Contrary to LPP, NLCP showed better range of external rotation especially at early postoperative period and more rigid fixation without metal failure. Authors presume LPP group to show better radiologic values and less operation time because of precontoured nature of LPP. MIPO technique using LPP could be considered as an easier and more accurate reduction method, but rigid fixation should be considered.

Cases of malalignment and malunion have been reported in fixation using indirect reduction and MIPO techniques [7–12] and thus precision in gap reduction is considered to be helpful in preventing such complications. In the current study, after we had failed to achieve bony union in initial 2 cases using LPP, we introduced a method to reduce fracture gaps using reduction clamps, followed by positional screw insertion to sustain the fracture-gap reduction. And there were no patients failed to achieve bony union afterwards.

Conventional open plating has longer operation time and high infection rates (around 3–7%) and thus less complicative surgical methods are need for surgeons. Iatrogenic radial nerve injuries due to extensive soft tissue dissection and radial nerve exposure in less than 5% of cases have also been reported [13–16]. However, MIPO technique does not need to open the fracture site. Its minimally invasive plate fixation through indirect reduction has fewer bleeding, lower infection rate, and less nonunion rate. MIPO technique has been developed for humeral shaft fractures to make stable fixation with less soft tissue dissection, thus improving healing rates and reducing complications such as infection and iatrogenic radial nerve palsy. MIPO has consistently shown healing rates of more than 90%, even in open fractures. Furthermore, iatrogenic radial nerve injury has only been reported in a single case from a pool of 114 fractures treated with MIPO [17].

Main indications of MIPO technique are long bone shaft fractures and metaphyseal fractures. Segmental and comminuted fractures can also be treated with indirect reduction. However, MIPO technique for spiral humerus shaft fractures with proximal or distal metaphyseal fracture extension is very difficult for fracture reduction and its maintenance. Patients who have undergone MIPO technique for humeral shaft fractures have good clinical outcomes and radiologic results [18, 19]. Malhan et al. have reported that 42 humeral shaft fracture patients with MIPO using a LCP have good improvement in DASH scores and correction of coronal and sagittal angulation [20]. The use of contoured locking plate and narrow LCP plate for treatment of these fractures is becoming popular [21–23]. Malal et al. [24] have reported that results of clinical scores such as VAS scale, DASH score, and Oxford Shoulder Score (OSS) was improved for 34 patients who had undergone fixation with long contoured locking plates for traumatic proximal humerus fractures with distal extension.

Previous study reported that MIPO showed early recovery of motion in adjacent joints for humeral shaft fractures. Median time to normal motion recovery has been reported to be 19 days in the shoulder and 60 days in the elbow [25]. Also, in our study, less external rotation in the NPP group at postoperative 3 and 6 months might be related to the proximal position of LPP at the early rehabilitation. However, there were no significant differences in shoulder ROM at the last follow up between the two groups.

Limitations of this study included the relatively small sample size and the relatively short term follow-up in both groups. Furthermore, due to the retrospective study design, neither group was perfectly randomized. A prospective randomized trial of larger volumes in both groups of long spiral humeral shaft fracture is needed to obtain more reliable results.

## Conclusion

For spiral humerus shaft fractures with or without metaphyseal fracture extension, both NLCP and LPP group could achieve satisfactory outcomes with complete bony union. However, the LPP group showed further correction of fracture angulation (AP and lateral X-ray) and shorter operation time, but, two metal failure was developed. MIPO technique using LPP could be considered an easier and more accurate reduction method for spiral humerus shaft fractures.

## Data Availability

The datasets generated and/or analysed during the current study that involve patient data are not publicly available because this was not established, nor would it have been accepted, as part of the ethics application. Other datasets and study material that do not require participant consent for sharing are publicly available and also available from the corresponding author on reasonable request.

## Abbreviations

Abd: Abduction
ASES: American Shoulder and Elbow Surgeons score
ER: External rotation
FF: Forward flexion
IR: Internal rotation
LCP: Locking compression plate
MRI: Magnetic resonance imaging
SST: Simple Shoulder Test score
UCLA: University of California at Los Angeles scale
